# Artificial Intelligence–Assisted Flexure and Landmark Identification Improves Reporting Completeness in Colonoscopy

**DOI:** 10.1055/a-2900-1202

**Published:** 2026-07-13

**Authors:** Radu-Alexandru Vulpoi, Ioannis Kafetzis, Mihaela Luca, Adrian Ciobanu, Oana Bogdana Barboi, Cristian Gheorghe, Eugen Dumitru, Alexander Meining, Alexander Hann, Vasile Liviu Drug

**Affiliations:** 1 Institute of Gastroenterology and Hepatology 37574 Grigore T. Popa University of Medicine and PharmacyIașiISRomania; 2 Interventional and Experimental Endoscopy (InExEn)Department of Internal Medicine 2 27207 University Hospital WürzburgWürzburgGermany; 3 Institute of Computer Science, Iasi Branch 54739 Romanian AcademyIaşiRomania; 4 Center of Gastroenterology and Hepatology 87267 University of Medicine and Pharmacy Carol Davila BucharestBucharestBucharestRomania; 5 Faculty of Medicine 112969 Ovidius University of ConstantaConstantaRomania

**Keywords:** endoscopy lower GI tract, colorectal cancer, CRC screening, quality and logistical aspects, image and data processing, documentation, quality management

## Abstract

**Introduction** Colorectal cancer is a major global health concern, with colonoscopy as the gold standard for detection and prevention. Accurate localization of findings remains challenging, limiting reporting objectivity and completeness, which can be supported by artificial intelligence (AI). We present an AI system that identifies the appendiceal orifice, ileocecal valve, and flexures, enabling automated colon segment localization.

**Methods** The AI was trained on 7264 manually annotated images from 991 patients and internally evaluated on 1238 images from 215 patients. Performance was assessed using accuracy, sensitivity, specificity, and F1-score. External validation was conducted on 78 public videos. The impact on standardized reporting was evaluated on 14 videos recorded in a different external clinic that did not contribute training data. These were independently evaluated by two expert endoscopists who reached a consensus that was used as the gold standard for flexure detection.

**Results** On the internal image test set, the AI achieved 94.5% accuracy, 76.7% sensitivity, 97.0% specificity, and 0.79 F1-score. In external validation on videos from a public dataset, the AI identified 91.7% of cecum segments and 66% of flexures, with 81.4% detected within 30 s of annotation. AI integration improved flexure identification in the external video dataset from the second clinic, increasing detected flexures by 53.8% and videos with both flexures identified by 133.3%.

**Conclusions** The AI reliably detects key anatomical landmarks, supporting colon segment localization. Its integration into reporting pipelines could enhance lesion localization, improve report completeness, and reduce documentation workload.

## Introduction

Colorectal cancer represents a major cause of cancer-related mortality, which can be significantly reduced through timely detection and prevention strategies, which includes colonoscopy. The primary findings during colonoscopy are colorectal polyps, which represent precancerous lesions. Removal of such lesions may prevent progression to colorectal cancer. Proper reporting of any findings and interventions undertaken during colonoscopy is therefore crucial. Complete and consistent documentation improves patient care, which is the reason behind the development of official reporting guidelines for colonoscopy. 1 2 Yet, it is common for endoscopic findings to be suboptimal reported. 3

AI has found numerous applications in medicine and, also in colonoscopy, where computer vision methods can be directly applied to the endoscopic image. The prime area of application was originally in polyp detection. 4 5 6 7 8 9 Moving on, the field broadened in methods for polyp characterization, 10 11 12 13 and size estimation. 14 15 16 17 Additionally, quality assessment of endoscopic images has been investigated. 18 19 Given its expanding application, the impact of AI on examiner confidence 20 and performance 21 has been investigated. The rapid development of AI for endoscopy is further supported by the increasing amount of available data. 22 Furthermore, automatic reporting for colonoscopy has been investigated in attempts to standardize reporting and reduce bureaucratic burden for physicians. 23 24

Automatic colonoscopy reporting begins with identification of the withdrawal phase of the colonoscopy. This can be achieved by recognizing key anatomical landmarks such as the appendiceal orifice and the ileocecal valve. A critical yet often overlooked factor is the precise localization of colonic findings. Distinguishing the five colonic regions (rectum, descending colon, transverse colon, ascending colon, and cecum) can be challenging. Owing to their distinct mucosal patterns, the rectum and cecum can usually be identified by visual inspection. In contrast, the descending, transverse, and ascending colon do not always present sufficiently distinct and consistent visual features for reliable segment differentiation based on endoscopic appearance alone. Accurate segmentation of these regions therefore still primarily relies on identifying the hepatic and splenic flexures, which serve as key anatomical landmarks. However, current automatic reporting approaches typically identify only the cecum, marking the beginning of withdrawal, without distinguishing other colonic segments. This limitation reduces their clinical impact, as the precise location of pathological findings is not reported.

In this work, we propose an AI to identify the appendiceal orifice, ileocecal valve, and flexures. This AI is intended to support automated reporting for colonoscopy by enabling localization of findings included in the report. 25 The present study therefore focuses on reporting related surrogate endpoints rather than direct clinical outcomes. The model was trained with data from multiple colonoscopies and validated in three settings. The first evaluation step was performed on a held-out set of image data distinct from the training data set. Subsequently, its performance was evaluated on a public dataset. Lastly, its ability to assist physicians in identifying the appendiceal orifice, ileocecal valve, and flexures in videos from another collaborating clinic that contributed no data for AI training was evaluated.

## Methods

### Data

Data for model training and internal testing was obtained from anonymized video recordings of endoscopic examinations. Identifying relevant images on videos is a cumbersome and time-consuming task, as it normally requires an expert to go through the examination video. Additionally, the proportion of relevant frames in a video is significantly lower compared to the irrelevant ones. To overcome this, we employed the active learning method proposed by Kafetzis et al. 26 to identify positive image examples of appendiceal orifice, ileocecal valve, and flexures among video frames. The examiner was also given the ability to navigate the video to collect either higher quality images or additional positive examples, by changing the angle or perspective. During the annotation process, hepatic and splenic flexures were collectively labeled as flexures rather than distinguished individually. This was done because the present study focused on frame-level visual detection of flexure appearance, whereas reliable differentiation between hepatic and splenic flexure generally requires temporal and procedural context from the withdrawal sequence. Such contextual modeling was not incorporated into the current approach. In total, 8502 images from 1206 patients from 5 centers were annotated. These images were split into training and testing on patient level, to avoid data leakage, ensuring training and testing data are completely distinct, following an 80–20 split. This resulted in 7264 images from 991 patients for training and 1238 images from 215 patients for testing. The data collected is analyzed in Table 1 . All examinations were recorded during a previous prospective study, 27 to which resection of colonic parts was an exclusion criterion. Therefore, no data from patients with resected parts of the colons were used during training of the AI.

**Table 1:** Data and label distribution in the overall, training, and testing data.

	Total	Training	Testing
Nr. Patients	1206	991	215
Nr. Images	8502	7264	1238
Appendiceal Orifice	981	840	141
Ileocecal Valve	893	759	134
Flexure	1434	1234	200

The proposed method was also evaluated on an external, publicly available dataset comprising 78 colonoscopy videos from the CAS-Colon dataset, 28 which includes annotations for various anatomical landmarks. For this study, we focused on cecum as well as hepatic and splenic flexures. To standardize evaluation, predictions corresponding to the appendiceal orifice and ileocecal valve were aggregated as the cecum because both landmarks are established indicators of cecal intubation and the external dataset provided cecum-level annotation for this region rather than separate labels for each structure. This approach also follows previous work by Kafetzis et al. 26 Annotations for the hepatic and splenic flexures were grouped together.

The selection of these specific anatomical landmarks was based on their clinical relevance for colonoscopy quality assessment and standardized reporting. 29 30 31 The appendiceal orifice and ileocecal valve were considered key markers of complete colonoscope intubation and of the initiation of the withdrawal examination sequence. 32 In contrast, the hepatic and splenic flexures were selected because they allow the colon to be divided into three clinically relevant segments: the right colon, the transverse colon, and the left colon, including the descending colon, sigmoid colon, and rectum. This segmentation mirrors the structure of the Boston Bowel Preparation Scale, 33 34 35 which evaluates bowel cleanliness across three colonic segments, with the flexures serving as anatomical transition points between these regions. Therefore, automatic recognition of these landmarks may support segment-based quality assessment and could be integrated with AI systems designed for automated Boston bowel preparation scoring. For example, Vulpoi et al. 18 developed an AI-based semantic segmentation system capable of quantifying, for each colonoscopy segment, the percentage of visible mucosa, residual content, artefacts, and lumen on a per-frame basis. However, the division of the colon into anatomical segments was still performed manually by expert reviewers. An AI system capable of automatically identifying the flexures could therefore enable automated segment allocation, contribute to a more objective and reproducible assessment of colonoscopy quality indicators, and potentially reduce the variability associated with human interpretation. 36

Finally, the potential of the AI to support landmark identification and standardized reporting was validated with 14 colonoscopy recordings from another endoscopy center that contributed no data to the training of AI. All recordings were obtained from patients without previous colorectal surgery. These videos were recorded using a different endoscopic processor compared to the ones used in the training data. Two board-certified gastroenterologists jointly reviewed the videos and annotated the time spans in which each label was clearly visualized. The final annotations were established by consensus rather than independent review.

### Description of the AI

A Class-Attention in Image Transformers (CaiT) model 37 was trained. The AI processed an endoscopic image as input. The output consisted of three values between 0 and 1, indicating the model’s confidence in the presence of the three target landmarks: flexure, appendiceal orifice, and ileocecal valve. These values were compared to the standard threshold value of 0.5 to obtain the final model prediction. A prediction of 1 indicates that the model is confident that the label is present in the image, whereas a label of 0 indicates the opposite. Preprocessing of the input included extraction of the endoscopic camera signal using the method implemented by Kafetzis et al., 38 rescaling to the size of 224 by 224, and normalization per color channel.

The proposed method predicts the presence of each label at the image level and subsequently aggregates these predictions over time to identify continuous segments within the video where each label is likely present following an approach similar to the one in the article by Kafetzis et al. 39

Further details of frame-level aggregation and temporal segmentation are provided in the Supplementary Material.

### Evaluation and Statistics

The performance of the AI model on the internal test dataset was assessed in terms of accuracy, sensitivity, specificity, and F1-score. These scores were calculated for each class individually and also averaged to represent the overall performance of the AI. Furthermore, confidence intervals for the metrics are obtained via bootstrapping. Furthermore, the ScoreCAM 40 explainability method was used to obtain insights behind the image parts that influence model predictions the most. During video validation, frame-by-frame AI predictions were collected for each examination recording. These predictions were aggregated to generate time spans containing frames where the AI is confident that each of the labels is present. Predictions were compared with the annotated segments. For the external validation on the CAS-Colon dataset, performance was assessed in terms of coverage, defined as the proportion of annotated segments with at least one overlapping AI prediction, and the number of additional AI predictions. Again, 95% confidence intervals were obtained using bootstrapping. Additionally, we quantified the number of annotated segments that either overlapped with a prediction or had a predicted segment occurring within 10 or 30 s of the annotated segment.

Finally, to evaluate the potential to support structured reporting on the 14 videos from a different external clinic, coverage and number of additional predicted segments were evaluated. Originally, two endoscopists collaboratively attempted to identify as many of the landmarks during the withdrawal phase in these recordings. Subsequently, the AI was used to obtain segment predictions for each landmark. Any predictions that did not overlap with the annotated ones were manually reviewed to assess if the corresponding label was indeed visible. If verified to contain the landmark, these segments were further used to assess the impact of the proposed method on increasing identification rate, with special attention given to cases where the landmark was entirely missed by the endoscopists in the original phase.

## Results

On the internal dataset consisting of 1238 images from 215 patients, the model achieved overall accuracy, sensitivity, specificity, and f1 score of 94.5% (95% CI: 93.7–95.2), 76.7% (95% CI: 72.8–80.5), 97.0% (95% CI: 96.4–97.6), and 0.79 (95% CI: 0.76–0.81), respectively, over all three labels. The values for each label are presented in Table 2 .

**Table 2:** Performance of the AI on the held-out testing dataset, per-label, and on average. Values in parentheses represent a 95% confidence interval. AI; Artificial Intelligence.

	Accuracy	Sensitivity	Specificity	F1-score
Appendiceal Orifice	96.4%	81.6%	98.3%	0.84
	(95.4–97.4)	(75.0–87.9)	(97.4–99.0)	(0.79–0.88)
Ileocecal Valve	95.5%	70.9%	98.5%	0.77
	(94.3–96.6)	(62.9–78.6)	(97.7–99.1)	(0.71–0.83)
Flexure (hepatic and splenic)	91.5%	77.5%	94.2%	0.75
	(90.0–93.1)	(72.2–83.0)	(92.8–95.7)	(0.7–0.79)

**Fig. 1:**
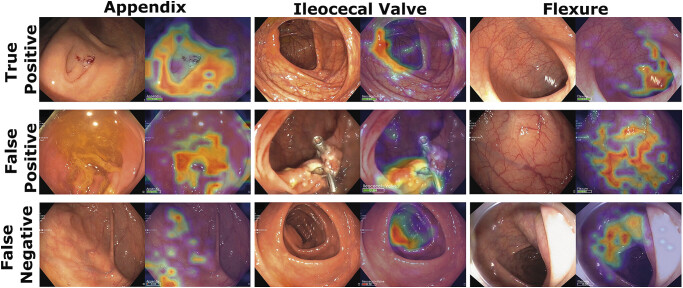
Correct and erroneous predictions of the AI for each class, along with explainability heatmaps indicating the most impactful parts of the input image.

**Fig. 2:**
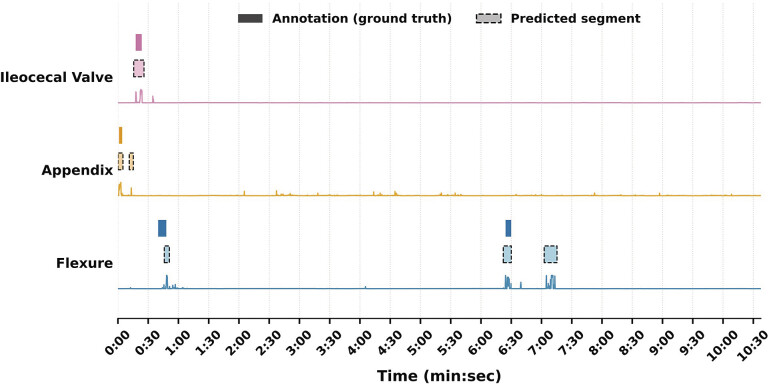
Visualization of the endoscopist annotated segments, model predicted segments, and per-frame model predictions for ileocecal valve, appendiceal orifice, and flexure. Timeline corresponds to the entire withdrawal phase of a colonoscopy video recording used as external validation.

Predictions of the AI were evaluated using the ScoreCAM method to identify the parts of the image having the biggest impact on model predictions. Examples of true positives, false positives, and false negatives for each label, along with the explainability heatmap, are presented in Fig. 1 , where the AI confidence is also visualized as a progress bar on the bottom left side of the image. The mean inference time per image was 0.021 s (95% CI: 0.020–0.022), which can be translated to FPS: 48 (95% CI: 44–51) frames-per-second. During external validation, AI-generated segments indicating identification of the three different landmarks were compared to the manually annotated ones ( Fig. 2 ).

In external validation on the CAS-Colon dataset, our AI accurately identified 91.7% (95% CI: 85.3–96.8) of cecum segments, with 95.5% (95% CI: 90.3–99.4) detected within 10 s and 96.8% (95% CI: 92.3–100) within 30 s of the annotated segment. The median number of additional predicted cecum segments per video was 2 (Q1–Q3: 1–3). For the hepatic and splenic flexures, the AI achieved 66% coverage (95% CI: 59–72.4), increasing to 72.4% (95% CI: 65.4–79.5) and 81.4% (95% CI: 75–86.4) within 10 and 30 s, respectively, with a median of 5 additional predicted segments per video (Q1–Q3: 2–7.7).

In the 14 external videos, physicians identified 22 clips where the appendiceal orifice was visualized, 21 (95.5%) of which had at least one overlapping AI segment. Similarly, for ileocecal valve, physicians identified 42 segments, 37 (88.1%) of which were also identified by the AI. Finally, for flexures, manual annotation identified 13 segments, 12 (92.3%) of which there was an overlapping prediction. Physicians identified the appendiceal orifice and the ileocecal in each video. Regarding the flexures, physicians identified both in 3 videos, at least one in 10 videos, and finally in 4 videos examiners failed to identify flexures.

The AI predicted additional segments, not overlapping with the ones annotated by the physicians, for all three labels. These segments were post-hoc evaluated to examine if they contained the corresponding labels. Out of the 32 additional segments identified by the AI as containing the appendiceal orifice, 15 (46.8%) of them were verified to contain it. Similarly, all 3 (100%) segments predicted with ileocecal valve contained it. Finally, for the 24 segments for flexures, 5 of them were caused by organ impressions in the cecum and were therefore discarded. Out of the remaining 19, 13 (68.4%) were indeed identified as containing flexures. Combining the flexures identified by the physicians with and without the help of AI, a total of 20 flexures (53.8% increase) were identified. This in turn increased the number of videos where both flexures were identified to 7, resulting in a 133.3% increase.

## Discussion

Accurate localization of findings during colonoscopy remains one of the key challenges for automated documentation systems. This can be addressed using an AI that identifies anatomical landmarks marking transitions between segments of the large bowel. This work introduces such an AI, which detects the appendiceal orifice, ileocecal valve, and flexures (splenic and hepatic). The appendiceal orifice and ileocecal valve are jointly used to define the cecum, as they consistently indicate this region at the onset of withdrawal. 26 Accordingly, during external validation, these predictions were aggregated into a single cecum category to match the external dataset’s annotation structure and enable direct comparison with the reference labels. The flexures enable localization of the ascending, transverse, and descending colon. This combined cecal designation also reflects standard reporting practice, in which findings in this region are typically described as cecal, sometimes with distance measurements in centimeters.

Performance of the AI was evaluated on images from a held-out patient cohort as well as on examination recordings from an external clinic. The held-out dataset consisted of 1238 images from 215 patients. The AI demonstrated high discriminative ability for all labels. Focusing on identification of flexures, the AI achieved an accuracy of 91.5% (95% CI: 90.0–93.1) and a high specificity of 94.2% (95% CI: 92.8–95.7) and satisfactory sensitivity of 77.5% (95% CI: 72.2–83.0). Similar results were obtained for ileocecal valve with accuracy, sensitivity, and specificity of 95.5% (95% CI: 94.3–96.6), 70.9% (95% CI: 62.9–78.6), and 98.5% (95% CI: 97.7–99.1) respectively. Finally, the results were even better regarding identification of the appendiceal orifice with 96.4% (95% CI: 95.4–97.4) accuracy, 81.6% (95% CI: 75.0–87.9) sensitivity, and 98.3% (95% CI: 97.4–99.0) specificity. Furthermore, the model was evaluated in terms of inference speed and was found to achieve close to 50 fps, which is in most cases was sufficient for real-time inference.

In external validation on the CAS-Colon dataset, our AI accurately identified 91.7% of cecum segments. Overall, 95.5% of annotated segments overlapped with a prediction or were within 10 s of a prediction, increasing to 96.8% within 30 s. AI predictions generated a median of only 2 additional segments per video (Q1–Q3: 1–3). Flexures were more challenging, with 66% coverage rising to 81.4% when considering prediction within 30 s and a median of 5 additional segments per video (Q1–Q3: 2–7.7). The relatively low number of extra predictions suggest that integrating this AI into a human–machine workflow would not overwhelm users with false positives, a common concern in colonoscopy, for example with polyp detection systems. 41 These results are particularly notable given by the moderate-to-low inter-annotator agreement reported for the dataset, especially regarding annotation of flexures, and the additional challenge posed by videos recorded using different endoscopic hardware. Overall, the findings highlight the potential of AI to support consistent and reliable landmark annotations.

Finally, the potential of the AI to support structured reporting was evaluated using 14 examination videos recorded at a different hospital. These analyses were designed to assess reporting-related surrogate endpoints, such as landmark identification and documentation support, rather than direct clinical benefit. The same videos were annotated by two gastroenterologists to indicate time intervals where each of the three labels of interest is present. The AI managed to identify 95.5% of the mark segments for appendiceal orifice, 88.1% of the mark segments for ileocecal valve, and 68.4% for possible flexures. Furthermore, using our AI predictions, the identified flexures increased by 53.8% (from 13 segments originally identified to 20 identified after AI predictions) and the number of examinations where both flexures were identified by 133.3%.

The problem of identifying anatomical landmarks in colonoscopy using AI has been investigated in the past. In a study by Che et al., 42 the authors identify the hepatic flexure, splenic flexure, and the sigmoid-descending colon junction. They train separate models for each label, which of course results in increased computational overhead. They describe high results with accuracy, precision, and recall of 90.7%, 90.9%, and 90.8%, respectively, for hepatic flexure and 90.8, 91.7, and 87.5 for splenic flexure, respectively. Yet, the high results can also be attributed to the fact that the images used for training and validation were taken from the same clips. Another similar work is by Kim et al. 43 where the authors use an AI in combination with density clustering to determine the cecum, flexures, and rectum. Their model achieves similar performance with ours in terms of flexure identification, having accuracy, sensitivity, and specificity of 79.6%, 81.2%, and 95.6%, respectively. The method proposed there has issues identifying the cecum, where the accuracy, sensitivity, and specificity are 84.6%, 83.2%, and 91.6%, respectively. Our results suggest that the proposed method, which emphasizes the identification of the appendiceal orifice and ileocecal valve, may achieve superior performance in recognizing the cecum. In the study by Tamhane et al., 44 the authors trained a Vision Transformer to identify the appendiceal orifice, ileocecal valve, and retroflexion in rectum, which achieved an overall accuracy of 81.8%, which is lower than the 94.5% achieved by our work. Another work focusing on automatic withdrawal time estimation 24 used similar landmarks to determine the beginning of withdrawal reported f1 scores for appendiceal orifice and ileocecal valve of 0.58 and 0.69, respectively, which are lower compared to those achieved with the proposed AI. The reported f1 scores for appendiceal orifice and ileocecal valve of 57.7 and 69.4, respectively, are lower compared to those achieved with the proposed AI.

Our study is also subject to limitations. The trained AI does not explicitly differentiate between hepatic and splenic flexure. As a result, the current model can support detection of anatomical transition points but cannot independently assign each detected flexure to a specific colonic segment. Such differentiation would likely require integration of temporal information from the withdrawal sequence, which was beyond the scope of the present study. An additional limitation is that the AI can sometimes misidentify colonic flexures due to impressions created by adjacent organs, particularly in the cecal region. Such false predictions can be addressed with simple rule-based corrections, for example by suppressing flexure activations in the cecum. Moreover, the AI relies on the visual appearance of the colon wall to identify flexures, meaning that advanced imaging modes that enhance the blue color channel could affect performance. However, these imaging modalities are easily recognizable in the endoscopic image, allowing clinicians to selectively ignore flexure predictions when such settings are used. An additional limitation is the absence of an independent external gold standard for evaluating the observed increase in flexure identification with AI assistance. This analysis relied on expert annotation and post hoc manual verification of additional AI-predicted segments and therefore remains subject to observer-dependent interpretation. Finally, the AI was evaluated on recorded data and video recordings, but not in clinical routine. A follow-up prospective study would be needed to properly evaluate the performance and contribution of the AI daily practice.

In conclusion, our work can reliably identify landmarks indicating the beginning of withdrawal in colonoscopy as well as flexures that may support segment-oriented documentation during the examination. This implies that the proposed method can be incorporated into automatic methods for colonoscopy. 39 Incorporation of the proposed method to such a pipeline would improve localization of the identified findings, significantly improving completeness and structure of the generated report. This in turn can reduce bureaucracy in the examination documentation process. However, the present study assessed performance in landmark detection and reporting support, rather than direct patient-related clinical outcomes. Furthermore, since the improvement in flexure identification when AI is involved, the impact of human–AI interaction should be investigated in the future to improve documentation accuracy and efficiency.

## Supplementary Material

Ergänzendes Material Supplementary Material
