# Supplementary material for: Artificial Intelligence–Assisted Flexure and Landmark Identification Improves Reporting Completeness in Colonoscopy
Source: Endosc Int Open. 2026 Jul 13;14:a29001202. doi: 10.1055/a-2900-1202 (PMC13460300; doi:10.1055/a-2900-1202)
Supplement: Supplementary file 1 [file 10-1055-a-2900-1202_29034654.pdf]

Supplementary Data: Artificial Intelligence–Assisted Flexure and Landmark Identification Improves Reporting Completeness in Colonoscopy

Radu-Alexandru Vulpoi, Ioannis Kafetzis, Mihaela Luca, Adrian Ciobanu, Oana-Bogdana Barboi, Cristian Gheorghe, Eugen Dumitru, Alexander Meining, Alexander Hann, & Vasile Liviu Drug

Frame-level aggregation and temporal segmentation.

The model produces frame-wise continuous prediction scores for each class (appendiceal orifice, ileocecal valve, and flexure), which are first temporally smoothed to reduce high-frequency noise. In this work, we apply a 1D convolutional moving average filter (kernel size = 5), yielding a smoothed probability for each frame.

Smoothed predictions are then thresholded at 0.5 to obtain a binary frame-level activation signal. To improve temporal consistency and reduce fragmentation, we apply a post-processing merging step using consecutive temporal convolutions over the binary sequence. Specifically, two sliding windows are used to fill short gaps and suppress isolated activations. In this work, the size of the sliding windows corresponded to the number of frames for 3 and 1 second, respectively. This procedure acts as a temporal tolerance mechanism, ensuring that brief interruptions in predictions do not result in separate detection intervals.

Final temporal segments are extracted by identifying contiguous positive regions in the merged signal and segments shorter than 1 second are discarded to reduce noisy detections. The resulting intervals represent the final frame-level aggregated predictions.

Endosc Int Open | DOI 10.1055/a-2900-1202 | © The Author(s).
